# *Notes from the Field:* Update on Pediatric Intracranial Infections — 19 States and the District of Columbia, January 2016–March 2023

**DOI:** 10.15585/mmwr.mm7222a5

**Published:** 2023-06-02

**Authors:** Emma K. Accorsi, Matt Hall, Adam L. Hersh, Samir S. Shah, Stephanie J. Schrag, Adam L. Cohen

**Affiliations:** ^1^Division of Bacterial Diseases, National Center for Immunization and Respiratory Diseases, CDC; ^2^Epidemic Intelligence Service, CDC; ^3^Children’s Hospital Association, Lenexa, Kansas; ^4^Department of Pediatrics, Division of Infectious Diseases, University of Utah, Salt Lake City, Utah; ^5^Cincinnati Children’s Hospital Medical Center, Cincinnati, Ohio.

In May 2022, CDC began an investigation of a possible increase in pediatric intracranial infections, particularly those caused by *Streptococcus* bacteria, during the preceding year (*1*). January 2016–May 2022 data from a large, geographically diverse network of children's hospitals showed altered patterns in pediatric intracranial infections after the onset of the COVID-19 pandemic (*1*). In this update, extended hospitalization data through March 2023 from 37 hospitals in 19 states and the District of Columbia showed a higher-than-expected number of pediatric intracranial infections beginning in August 2021, with a large peak during winter 2022–2023. Pediatric intracranial infections are recognized as a severe complication of viral respiratory infection and sinusitis (*2*), and the winter 2022–2023 peak coincided with spikes in respiratory virus circulation*^,^^†^ (*3*,*4*). Even during this peak, intracranial infections remained rare. CDC continues to track trends in pediatric intracranial infections and recommends that all persons aged ≤18 years remain current with recommended vaccinations, including influenza and COVID-19.^§^

To characterize national trends in pediatric intracranial infections, CDC analyzed pediatric hospitalizations for brain abscesses, epidural empyemas, and subdural empyemas reported to the Children’s Hospital Association’s Pediatric Health Information System (PHIS) by 37 tertiary referral children’s hospitals in 19 states and the District of Columbia. The included hospitals consistently reported to PHIS during January 1, 2016–March 31, 2023 (the most recent data available when the analysis was performed).^¶^ All inpatient encounters with persons aged ≤18 years that had a primary or secondary *International Classification of Diseases, Tenth Revision, Clinical Modification* discharge diagnosis code G06.0 (intracranial abscess and granuloma) or G06.2 (extradural and subdural abscess, unspecified) during the study period were included. Because the study period was extended from that of the earlier report (*1*), the subset of included hospitals differed slightly from that previously analyzed and reported. Data were analyzed in aggregate and by U.S. Census Bureau region (Northeast, Midwest, South, and West) using R software (version 4.0.3; R Foundation) with RStudio (version 1.3.1093; Posit, PBC). This activity was reviewed by CDC and was conducted consistent with applicable federal law and CDC policy.**

Using pediatric intracranial infection hospitalization data collected during 2016–2019, the monthly median (34; IQR = 29.75–42.00) and maximum (61) number of cases were calculated as a prepandemic baseline (Figure). After the onset of the COVID-19 pandemic in March 2020, monthly intracranial infection case counts remained below the baseline median during May 2020–May 2021. Monthly case counts exceeded the median during August 2021–March 2023^††^ but did not exceed the baseline maximum until a large peak (102 cases) in December 2022. During January–March 2023, case counts began to decline but remained above the baseline maximum. Although some variability between U.S. Census Bureau regions was observed, overall patterns were generally similar: consistently low case counts after the onset of the pandemic, then a period of increase beginning in mid- to late 2021 followed by a large peak during winter 2022–2023 (Figure). Demographic characteristics of patients (age, race and ethnicity, and sex), measures of severity (length of hospitalization, intensive care unit admission, and in-hospital mortality), and the percentage of patients with a complex chronic condition (*5*) remained approximately stable over the study period and were similar to values reported previously (*1*).

**FIGURE:**
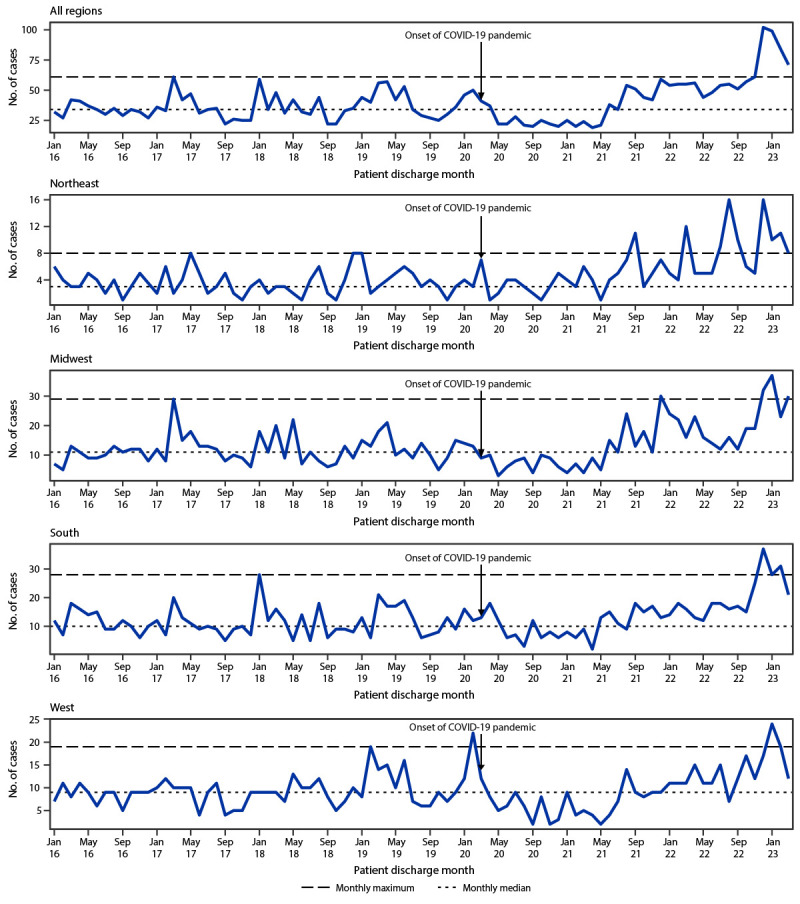
Cases of intracranial infection* among persons aged ≤18 years by U.S. Census Bureau region — Pediatric Health Information System, 19 states and the District of Columbia, January 2016–March 2023^†^ * The median and maximum number of cases per month during 2016–2019, by U.S. Census Bureau region. ^†^ Data from 37 children’s hospitals in 19 states and the District of Columbia. The number of hospitals that provided data in each U.S. Census Bureau region were as follows: five (Northeast Region), 13 (Midwest Region), 11 (South Region), and eight (West Region).

This analysis in a large, geographically diverse network of children's hospitals showed elevations in pediatric intracranial infections beginning in mid-2021 with a large spike in winter 2022–2023, both nationally and by U.S. Census Bureau region. Despite these observed increases, pediatric intracranial infections remain rare. These infections are often preceded by viral respiratory infection and sinusitis, and recent trends might be driven by concurrent, heightened pediatric respiratory pathogen transmission (*3*,*4*). All persons aged ≤18 years should be up to date with recommended vaccinations, including influenza and COVID-19. CDC will continue to track trends in pediatric intracranial infections.

## Footnotes

*
https://www.cdc.gov/rsv/research/rsv-net/dashboard.html

†
https://gis.cdc.gov/GRASP/Fluview/PedFluDeath.html

§
https://www.cdc.gov/vaccines/schedules/hcp/imz/child-adolescent.html

¶ Population denominators were not available; therefore, analysis was limited to hospitals that reported data for each month during the study period.

** 45 C.F.R. part 46.102(l)(2), 21 C.F.R. part 56; 42 U.S.C. Sect. 241(d); 5 U.S.C. Sect. 552a; 44 U.S.C. Sect. 3501 et seq.

†† During March 2020–May 2022, as described, the current findings were not identical to those previously reported because of variability in the hospitals included in each analysis. In the current analysis with an extended period of observation, a decline was observed in May 2022, but not to the median value. In the earlier analysis, cases were below the median during April 2020–June 2021, above the median during July 2021–April 2022, and declined to the median in May 2022.
